# Rapid Point-Of-Care Breath Test for Biomarkers of Breast Cancer and Abnormal Mammograms

**DOI:** 10.1371/journal.pone.0090226

**Published:** 2014-03-05

**Authors:** Michael Phillips, J. David Beatty, Renee N. Cataneo, Jan Huston, Peter D. Kaplan, Roy I. Lalisang, Philippe Lambin, Marc B. I. Lobbes, Mayur Mundada, Nadine Pappas, Urvish Patel

**Affiliations:** 1 Breath Research Laboratory, Menssana Research Inc., Newark, New Jersey, United States of America; 2 Department of Medicine, New York Medical College, Valhalla, New York, United States of America; 3 Swedish Cancer Institute, Seattle, Washington, United States of America; 4 HackensackUMC Mountainside, Montclair, New Jersey, United States of America; 5 Division of Medical Oncology, Department of Internal Medicine, GROW School of Oncology and Developmental Biology, Maastricht University Medical Centre, Maastricht, The Netherlands; 6 Department of Radiotherapy (MAASTRO), GROW School of Oncology and Developmental Biology, Maastricht University Medical Centre, Maastricht, The Netherlands; 7 Department of Radiology, GROW School of Oncology and Developmental Biology, Maastricht University Medical Centre, Maastricht, The Netherlands; 8 Saint Michael's Medical Center, Newark, New Jersey, United States of America; Health Canada and University of Ottawa, Canada

## Abstract

**Background:**

Previous studies have reported volatile organic compounds (VOCs) in breath as biomarkers of breast cancer and abnormal mammograms, apparently resulting from increased oxidative stress and cytochrome p450 induction. We evaluated a six-minute point-of-care breath test for VOC biomarkers in women screened for breast cancer at centers in the USA and the Netherlands.

**Methods:**

244 women had a screening mammogram (93/37 normal/abnormal) or a breast biopsy (cancer/no cancer 35/79). A mobile point-of-care system collected and concentrated breath and air VOCs for analysis with gas chromatography and surface acoustic wave detection. Chromatograms were segmented into a time series of alveolar gradients (breath minus room air). Segmental alveolar gradients were ranked as candidate biomarkers by C-statistic value (area under curve [AUC] of receiver operating characteristic [ROC] curve). Multivariate predictive algorithms were constructed employing significant biomarkers identified with multiple Monte Carlo simulations and cross validated with a leave-one-out (LOO) procedure.

**Results:**

Performance of breath biomarker algorithms was determined in three groups: breast cancer on biopsy versus normal screening mammograms (81.8% sensitivity, 70.0% specificity, accuracy 79% (73% on LOO) [C-statistic value], negative predictive value 99.9%); normal versus abnormal screening mammograms (86.5% sensitivity, 66.7% specificity, accuracy 83%, 62% on LOO); and cancer versus no cancer on breast biopsy (75.8% sensitivity, 74.0% specificity, accuracy 78%, 67% on LOO).

**Conclusions:**

A pilot study of a six-minute point-of-care breath test for volatile biomarkers accurately identified women with breast cancer and with abnormal mammograms. Breath testing could potentially reduce the number of needless mammograms without loss of diagnostic sensitivity.

## Introduction

Breast cancer is the most commonly diagnosed cancer in women, in whom it is second only to lung cancer as a cause of cancer death [1]. The National Cancer Institute estimated that more than 232,000 US women would be diagnosed with breast cancer in 2013 and nearly 40,000 will die of the disease [2]. In order to reduce the number of breast cancer deaths, many countries have established screening mammography programs to detect and treat early-stage disease [3]. However, screening mammography may be associated with an increased risk of radiation-induced breast cancer, as well as with overdiagnosis and overtreatment [4], [5]. Many women decide not to take the test even when it is readily available, and screening mammography is frequently underutilized. Several factors may influence this decision, including fear of pain and radiation exposure, as well as ethnicity, poverty, and level of education [6]–[9].

These limitations of screening mammography have stimulated the search for new tools to identify early-stage breast cancer without any discomfort or risk [10]. Members of our group have previously reported a breath test for volatile organic compounds (VOCs) employing gas chromatography (GC) and mass spectrometry (MS) that identified sensitive and specific biomarkers of breast cancer. Multivariate models containing as few as five breath VOCs accurately predicted the presence or absence of breast cancer [11]–[13]. Other researchers have also reported evidence of breath VOC biomarkers of breast cancer employing a variety of different approaches including GC MS [14]–[16], exhaled breath condensate [17], electronic noses [18]–[21], and sniffing dogs [22], [23].

In recent years, the clinical value of breath testing has been increasingly recognized in other applications such as urea breath tests for detection of Helicobacter pylori infection and nitric oxide breath concentrations for monitoring the severity of bronchial asthma [24]. Clinical studies have also demonstrated breath VOC biomarkers in other diseases, including lung cancer [25], [26], pulmonary tuberculosis [27], [28], radiation exposure [29], and heart transplant rejection [30].

The objective of this study was to test the hypothesis that a rapid point-of-care breath test could detect breath biomarkers of breast cancer. We tested this hypothesis in a multicenter clinical study with the BreathLink system that collects, concentrates, and assays breath VOCs in approximately six minutes. The BreathLink system was previously reported to identify breath biomarkers of active pulmonary tuberculosis [31].

## Materials and Methods

### Clinical sites

Three breast cancer treatment centers participated in the study: Saint Michael's Medical Center, Newark, NJ, Swedish Medical Center, Seattle, WA, and Maastricht University Medical Center, Maastricht, the Netherlands.

### IRB approval and informed consent

The Institutional Review Board (IRB) at all collaborating sites approved the research. All subjects gave their signed informed consent to participate.

### Human subjects

Two groups of subjects were studied.

#### a. Normal healthy women attending for a screening mammogram

Subjects were included if they were female, aged > = 18 years, understood the study, were willing and able to give signed informed consent to participate, and could donate a breath sample during 7-day period prior to screening mammography. Subjects were excluded if they had a previous history of breast cancer, cancer at any other site, breast biopsy, abnormal mammogram or palpable breast mass. The screening population at Maastricht University with a negative mammogram including a subset of women with increased risk for developing breast cancer because of BRCA 1 or 2 positivity, or high incidence of breast cancer in the family without known mutation.

#### b. Women with an abnormal screening mammogram referred for breast biopsy

Subjects were included if they were female, aged > = 18 years, understood the study, were willing and able to give signed informed consent to participate, and had been referred for breast biopsy because of an abnormal screening mammogram (BIRADS 3–5) or a palpable breast mass.

### Point-of-care breath test

The BreathLink system been described previously [31]. In summary, subjects wore a nose-clip and respired normally for 2.0 min, inspiring room air from a valved mouthpiece, and expiring into a breath reservoir. A microbial filter prevented contamination of the reservoir (efficiency >99.9% for bacteria and >99.8% for viruses) (Vital Signs, Totowa, NJ). The mouthpiece and filter were discarded after a single use; they presented low resistance to expiration, so that subjects could donate breath samples without effort or discomfort. Alveolar breath VOCs were pumped from the breath reservoir through a sorbent trap where they were captured and concentrated. VOCs in a similar volume of room air were separately collected and concentrated in the same fashion. Breath VOCs were analyzed with a portable gas chromatograph (GC) coupled to a surface acoustic wave (SAW) detector. The analyzer was calibrated daily with an external standard, a mixture of C6 to C22 n-alkanes (Restek Corporation, Bellefonte, PA 16823, USA). Each breath test comprised collection and analysis of separate samples of breath and room air. The time from commencement of breath collection to completion of GC analysis was six minutes. Files containing anonymized chromatographic data and electronic case report forms were stored locally on the BreathLink, then encrypted and transmitted via internet to a server at the Menssana Research Breath Research laboratory in Newark, NJ, USA, where they were decrypted and stored for analysis.

### Analysis of data

Chromatograms and results of mammograms and biopsies were transmitted to Schmitt & Associates, Newark, NJ, who analyzed all data independently without any participation by the study sponsors. The principles of the method have been described in reference [32]. In summary, each chromatogram was converted into a series of data points derived from the SAW detector signal (3013 scans/min) The alveolar gradient (i.e. abundance in alveolar breath minus abundance in ambient room air) was then determined [31].

### Identification of biomarkers and construction of predictive algorithm

Biomarkers were identified and algorithms were developed independently in three groups: women with breast cancer on biopsy compared to women with normal screening mammograms, normal versus abnormal screening mammograms (BIRADS 1–2 versus BIRADS 3–6), and cancer versus no cancer on breast biopsy. The methods have been described previously [32]. In summary, chromatograms were permuted using multiple Monte Carlo simulations to identify the chromatographic peak segments that identified disease with greater than random accuracy. The alveolar gradients of all chromatographic peak segments were compared in disease and control groups and ranked as candidate biomarkers according to their C-statistic values i.e. the AUC of the receiver operating characteristic (ROC) curve [33]. The underlying statistical distribution of each chromatographic peak segment was determined with multiple Monte Carlo simulations by randomly permuting subjects between disease and control groups, and performing 40 estimates of the C-statistic value. Differences between the C-statistic values obtained with correct diagnosis and statistic obtained with random permutations of diagnosis identified the chromatographic peak segments that were apparent biomarkers, because they identified the disease group with better than random accuracy [34], [35]. The chromatographic peak segments identified as biomarkers of disease were employed to construct a multivariate predictive algorithm with weighted digital analysis (WDA) [36].

### Cross-validation of predicted outcomes

A repeated leave-one-out (LOO) bootstrap method was employed in order to provide a robust and conservative estimate of prediction error [37]. One subject at a time was removed from the total data set, and the remaining data set was used to derive an algorithm employing multiple Monte Carlo simulations and WDA as described above. The left-out subject was used to calculate the discriminant value based on the algorithm, and results from all subjects were combined in a ROC curve

## Results

### Human subjects

244 subjects fulfilled recruitment criteria and provided technically usable breath chromatograms. Their characteristics are shown in Table 1. No adverse effects of the breath test were reported.

**Table 1:** Patient characteristics.

***Screening mammography group (n = 130)***	
Normal (BIRADS 1–2)	93
Abnormal (BIRADS 3–6)	37
***Breast biopsy group (n = 114)***	
***Cancer negative***	**79**
***Cancer positive***	**35**
Invasive ductal carcinoma	20
Ductal carcinoma in situ	8
Invasive lobular carcinoma	5
Sarcomatoid carcinoma	1
Atypical papillary lesion	1

#### Identification of biomarkers and construction of predictive algorithms

Following correction for multiple signals from a single biomarker generated by adjacent SAW detector scans, fewer than ten significant biomarkers were detected in each comparison group.

#### Women with breast cancer on biopsy versus women with normal screening mammograms (BIRADS 1–2 versus BIRADS 3–6)

**Figure 1:**
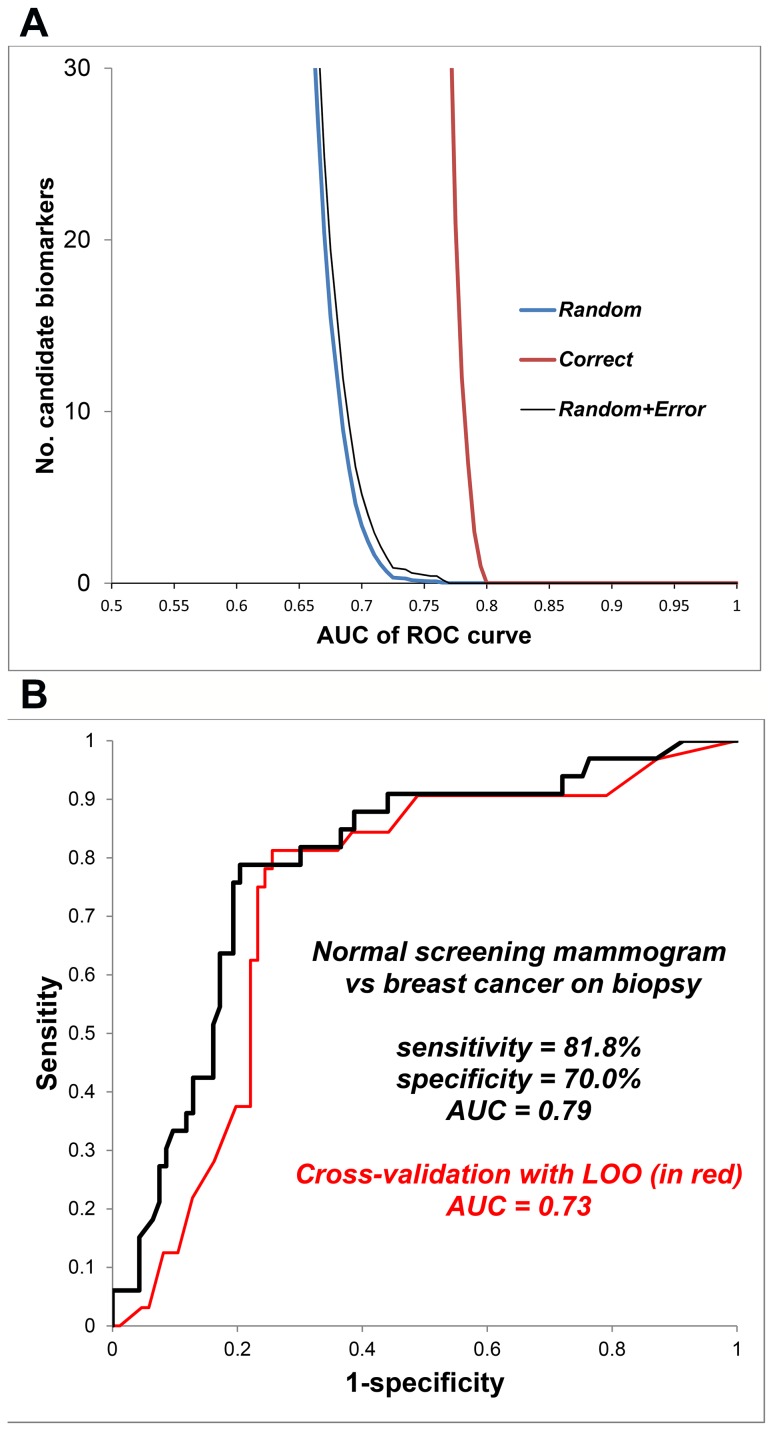
Breath test outcome in healthy women (normal screening mammogram) versus women with breast biopsy positive for cancer. *Identification of breath biomarkers (upper panel):* A list of candidate breath biomarkers of disease was obtained by segmenting chromatograms into a time series of alveolar gradients, where the alveolar gradient comprised detector response in breath minus corresponding detector response in room air. The diagnostic accuracy of each candidate biomarker was quantified as the area under curve (AUC) of its associated receiver operating characteristic (ROC) curve. This figure displays the number of candidate biomarkers (y-axis) as a function of their diagnostic accuracy (x-axis). The “correct” curve employed the correct assignment of diagnosis (normal mammogram or cancer on biopsy). The “random” curve employed multiple Monte Carlo simulations comprising 40 random assignments of diagnosis in order to determine the random behavior of each candidate biomarker. The horizontal separation between the “correct” and “random” curves varies with the amount of diagnostic information in the breath signal. Where the number in the “random” curve declines to <1, its vertical distance from the “correct” curve identifies the excess number of candidate biomarkers that identified the disease group with greater than random accuracy. The number of apparent biomarkers with greater than random accuracy exceeded 30, but several segments were closely adjacent in the time series, consistent with approximately 10 biomarker peaks in the chromatogram. Similar analyses were also performed in normal versus abnormal screening mammograms and cancer versus no cancer on breast biopsy in order to develop separate algorithms. ***Diagnostic accuracy of the breath test (lower panel):*** The ROC curve displays the breath test's accuracy in distinguishing healthy women with a normal screening mammogram from women whose breast biopsy was positive for cancer. The breath test employed a multivariate predictive algorithm derived from the biomarkers with greater than random accuracy that were identified with the Monte Carlo simulations in the left panel. Sensitivity and specificity values were determined from the point on the ROC curve where their sum was maximal. Cross-validation of predicted outcomes is shown in red ROC curve. A repeated leave-one-out bootstrap method was employed to estimate the prediction error (method described in text).

Figure 1 displays outcomes of Monte Carlo simulations in upper panel, and the performance of breath biomarker algorithms in the middle panel: 81.8% sensitivity, 70.0% specificity, 79% accuracy (C-statistic value. Cross-validation of predicted outcomes in the lower panel displays LOO ROC curve with AUC = 0.73.

#### Women with normal versus abnormal screening mammograms

**Figure 2:**
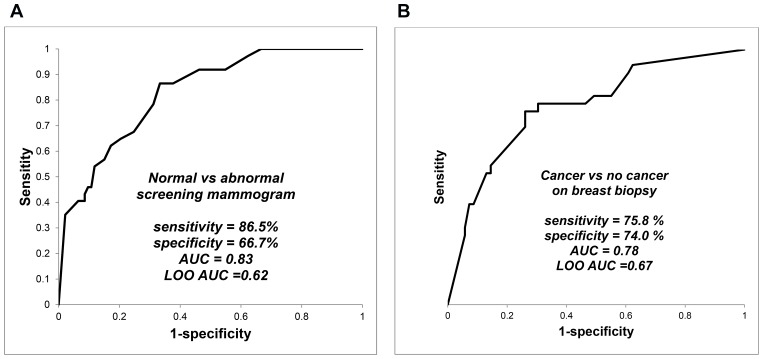
Breath test outcomes in screening mammography and in breast biopsy. Identification of breath biomarkers, determination of diagnostic accuracy of the breath test, and LOO cross-validation of predicted outcomes were performed in the same fashion as described in Figure 1. Sensitivity and specificity values were determined from the point on the ROC curve where their sum was maximal. The left panel displays comparison of women with normal and abnormal screening mammograms, and the right panel displays comparison of women with cancer and no cancer on breast biopsy.

Figure 2 (left panel) displays ROC curve with sensitivity = 86.5%, specificity = 66.7%, and AUC = 0.83; LOO ROC curve AUC was 0.62.

#### Women with cancer versus no cancer on breast biopsy

Figure 2 (right panel) displays ROC curve with sensitivity = 75.8%, specificity = specificity = 74.0% and AUC  = 0.78; LOO ROC curve AUC was 0. 0.67.

#### Expected outcome of screening

Table 2 shows the expected outcome of screening one US million women for breast cancer with the breath test. These predictions employ the following assumptions: test sensitivity = 81.8%, specificity  = 70.0% (Figure 1) and prevalence of 3.95 breast cancers per 1000 screening mammograms [38].

**Table 2:** Expected outcome of screening for breast cancer with breath test.

		*CANCER STATUS*					
		*Positive*	*Negative*		*Pre-test value (%)*	*Post-test value (%)*	*Enrichment factor*
***TEST***	***positive***	TP = 3,231	FP = 298,815	***PPV***	0.395	1.070	2.708
***RESULT***	***negative***	FN = 719	TN = 697,235	***NPV***	99.605	99.897	1.003
	***Total***	3,950	996,050				

The table summarizes the expected outcome of screening one million women in the USA with a breath test, assuming test sensitivity = 81.8% and specificity = 70.0% (Figure 1, middle panel), and prevalence of breast cancer is 3.95 cancers per 1000 (based on mean cancer detection rate of screening mammography) [44]. TP = true positives, FP = false positives, FN = false negatives, and TN = true negatives. PPV and NPV are positive and negative predictive value respectively, where PPV = TP/(TP+FP), NPV = TN/(TN+FN), and enrichment factor = post-test value/pre-test value.

## Discussion

This study tested the hypothesis that a rapid point-of-care breath test could detect breath biomarkers of breast cancer. The main finding in the study group was that the test identified women with breast cancer with 79% accuracy. The breath test also distinguished between normal and abnormal mammograms with 83% accuracy and between breast biopsies read as positive or negative for cancer with 78% accuracy.

The hypothesis was supported by the consistency of these findings with previous reports that laboratory-based breath tests employing GC MS identified breath biomarkers of breast cancer and abnormal mammograms [11]–[13]. The accuracy of rapid point-of-care breath testing was comparable to screening mammography with digital or film imaging (78% and 74% accuracy respectively) [39]. The fundamental point of difference between the tests is that mammography detects altered anatomy, while breath testing detects altered biochemistry.

The hypothesis was further supported by its rational basis in biology. Breath biomarkers of breast cancer have been linked to increased oxidative stress in breast cancer tissue, where an activated phenotype in stromal fibroblasts causes increased secretion of hydrogen peroxide, a powerful oxidant [40]. Oxidative stress is accompanied by increased production of reactive oxygen species that peroxidate polyunsaturated fatty acids in cell membranes, liberating alkanes such as pentane and alkane derivatives that are expired in the breath [15] [41], [42]. In addition, the abundance of VOCs in breath may be modulated by induction of polymorphic cytochrome p450 mixed oxidase such as CYP2E1 enzymes [43]. Breast cancer is accompanied by upregulation of several cytochrome p450 enzymes including aromatase, which increases the tissue concentration of estradiol and activates a large number of carcinogenic genes via estrogen receptor-alpha in malignant epithelial cells [44]. These upregulated cytochrome p450 enzymes may also accelerate the degradation of circulating endogenous VOCs, causing detectable changes in the composition of breath. The effect of the size or the stage of the tumor was not analyzed in this report because the total number of cancers was too small to permit statistically meaningful stratification of the data.

Our previous reports of breath biomarkers of breast cancer identified candidate VOCs with GC MS. The main VOC biomarkers included alkanes and alkane derivatives e.g. tridecane, tetradecane, and dodecane, 2,7,10-trimethyl, all of which were consistent with downstream products of lipid peroxidation induced by oxidative stress [13]. The candidate biomarkers also included a number of benzene derivatives whose origin is not known. This study employed a rapid point-of-care breath test employing gas chromatography with a surface acoustic wave detector (GC SAW) that was previously reported to identify breath biomarkers of active pulmonary tuberculosis [31]. The main advantage of GC SAW over GC MS is its suitability for point-of-care breath testing because of its rapidity, robustness, ease of use, and lower cost. However, the SAW detector responds only to the mass of VOCs in breath and unlike MS, does not provide information about their chemical structure. Future studies will focus on determination of the Kovats Indices (i.e. relative chromatographic retention times) of the breath VOC biomarkers observed with GC SAW, in order to correlate biomarkers with those observed using GC MS.

As Table 3 illustrates, other researchers have also reported breath VOC biomarkers of breast cancer consistent with products of oxidative stress e.g. pentane. Where other breath VOC biomarkers of breast cancer have been reported, these differences may have arisen in part from an artifact of analysis with1D GC MS. Breath VOC separation with a newer technique, 2-dimensional GC, resolves ten times as many peaks as 1D GC i.e. 2,000 VOCs in a sample versus 200 [45]. It is likely that many apparently “pure” breath VOC biomarkers that were previously observed with 1D GC MS in breath were actually co-eluting mixtures of several different VOCs e.g. breath hexane observed with 1D GC MS was resolved by 2-dimensional GC into a mixture of seven different VOCs that included hexane and six other VOCs. As a consequence, previous studies employing 1D GC MS may have erroneously reported the chemical structure of apparent biomarkers of breast cancer.

**Table 3:** Summary of previous reports by other investigators of breath biomarkers of breast cancer.

*Author #1*	*Assay method*	*Outcome*
Hietanen [15]	GC	Increased pentane in breast cancer
Mangler [14]	GC MS	Specific pattern of 5 VOCs in breast cancer: 3-methylhexane, decene, caryophyllene, naphthalene, and trichloroethylene
Patterson [16]	GC MS	Clustering patterns in 383 VOCs classified breast cancer with 77% accuracy; 72% sensitivity, 64% specificity
Peng [20]	Nanosensor array And GC MS	Differentiated between ‘healthy’ and ‘cancerous’ breath and different cancer types
Stolarek [17]	Fluorimetry	Increased H2O2 level in exhaled breath condensate in breast cancer
McCulloch [22]	Sniffing dogs	Detected breast cancer with sensitivity 88% and specificity 98%
Shuster [18]	Nanosensor array	Statistically significant differences between benign and malignant breast conditions
Xu [21]	Nanosensor array	Detected breast cancer with four VOCs: heptanal, acetophenone, isopropyl myristate and 2-propanol.

Where assay techniques were employed that separated VOCs with gas chromatography (GC) and identified them with mass spectrometry (MS), a number of breath VOC biomarkers were consistent with products of oxidative stress e.g. pentane, hydrogen peroxide, and alkane derivatives including heptanal and propanol.

Modern breath assays with highly selective technologies can detect more than 2,000 different VOCs in a single sample of breath, providing a powerful new tool for biomarker discovery [29], [45]. However, this benefit has been accompanied by an increased statistical risk of mistakenly identifying spurious biomarkers amongst a large number of candidates. This problem has generated some picturesque metaphors in the breath research community: “Finding the needles in a haystack” can yield “voodoo biomarkers” by “seeing faces in the clouds”. In order to minimize this problem, a predictive multivariate algorithm should first, be derived in a training set employing only those biomarkers identified with high statistical significance, and second, the algorithm should be validated in an independent predictive set. In this comparatively small pilot study, the multiple Monte Carlo simulations identified a statistically significant set of candidate biomarkers that were cross-validated with a repeated leave-one-out bootstrap method. However, these findings will require validation in larger future studies.

The major technical challenge to screening programs for breast cancer is the low prevalence of disease in populations at average risk, and the resulting low yield of true-positive findings. In a study of more than two million screening mammograms, the mean cancer detection rate for individual radiologists was 3.95 cancers per 1000 examinations [38] i.e. the pre-test a priori likelihood of finding cancer on a screening mammogram was only 1 in every 250 tests. The results of this study suggest that that a positive result on a breath test can enrich the screened population nearly threefold for risk of breast cancer, while a negative result could safely exclude 99.9% of all women with the disease. This could dramatically reduce needless exposure to radiation, because the overwhelming majority of screened women are cancer-free (99.61%), but they are exposed to the risks and discomforts of mammography in order to learn that their test result was negative. A cancer-negative mammogram is a needless procedure, but this can only be known after the event.

Breath biomarkers in this study group identified abnormal screening mammograms with 100% sensitivity and 33% specificity (Figure 2). If this finding is confirmed in validation studies, a breath test could potentially identify all women who will have an abnormal screening mammogram while safely excluding a third of population who will have a normal result and do not require further testing. However, further studies will be required to confirm these findings in larger numbers of subjects.

In addition, the chromatographic peak segments that were identified as biomarkers of disease contained no information about the chemical structure of the VOCs detected, since the SAW detector responded only to mass of eluting compounds. Another goal of future studies will be to determined the Kovats Index values of these biomarkers by comparing their retention times to those of known n-alkane standards, and comparing them to the Kovats Index values of VOCs whose structures have been identified by mass spectrometry.

We conclude that a rapid point-of-care test for biomarkers in breath accurately identified women with breast cancer and with abnormal mammograms, and distinguished between breast biopsies read as positive or negative for cancer. These findings of a comparatively small pilot study should be interpreted with caution, and will require validation in larger studies. However, if these findings are confirmed in future studies, breath testing could be employed as a complementary procedure ancillary to mammography with the potential to reduce the number of needless procedures and reduce the costs of discovering new cases of breast cancer.
